# Patients’ views on using human embryonic stem cells to treat Parkinson’s disease: an interview study

**DOI:** 10.1186/s12910-022-00840-6

**Published:** 2022-10-19

**Authors:** Jennifer Drevin, Dag Nyholm, Håkan Widner, Trinette Van Vliet, Jennifer Viberg Johansson, Elena Jiltsova, Mats Hansson

**Affiliations:** 1 Centre for Research Ethics and Bioethics, Uppsala University, Box 564, 751 22 Uppsala, Sweden; 2 Department of Medical Sciences, Uppsala University Hospital, Uppsala University, 751 85 Uppsala, Sweden; 3 Neurology Clinic, Skåne University Hospital, 221 85 Lund, Sweden; 4 The Institute for Future Studies, Holländargatan 13, 111 36 Stockholm, Sweden

**Keywords:** hESC, ATMP, Human embryonic stem cells, Advanced therapy medicinal product, Parkinson’s disease, Ethics

## Abstract

**Background:**

Human embryonic stem cells (hESC) as a source for the development of advanced therapy medicinal products are considered for treatment of Parkinson’s disease (PD). Research has shown promising results and opened an avenue of great importance for patients who currently lack a disease modifying therapy. The use of hESC has given rise to moral concerns and been the focus of often heated debates on the moral status of human embryos. Approval for marketing is still pending.

**Objective:**

To Investigate the perspectives and concerns of patients with PD, patients being the directly concerned stakeholders in the ethical discussion.

**Methods:**

Qualitative semi-structured interviews related to this new therapy in seventeen patients from two Swedish cities.

**Results:**

The participants expressed various interests related to the use of human embryos for development of medicinal therapies; however, overall, they were positive towards the use of hESC for treatment of PD. It was deemed important that the donating woman or couple made the choice to donate embryos voluntarily. Furthermore, there were concerns that the industry does not always prioritise the patient over profit; thus, transparency was seen as important.

## Background

### Human embryonic stem cells

Human embryonic stem cells (hESC) are undifferentiated, pluripotent cells derived from the inner cell mass of a human blastocyst at approximately less than a week. Long before the first hESC lines were generated in the 1990s [1], researchers had seen the potential benefit of using hESC from embryos in the pre-implantation phase in regenerative medicine. The abilities of hESC to differentiate into any specialised cell, together with their capacity to self-renew, have raised hopes of them being able to replace damaged cells and tissues, and being useful in the treatment of various diseases.

Definitions used: Embyo—the early stage of development of an organism. For humans, the term encompasses the period from fertilisation to the end of organ formation, at week 9 after conception.

The embryonic period is further divided into one pre-implantation phase up to 5 days after fertilisation, when the embryo can be held in culture, or kept frozen.

Pluripotent embryonic stem cells develop in the inner cell layer of a blastocyst and can be retrieved in this phase.

After implantation into the uterus, embryos are generally not considered for medicinal purposes.

An embryo has no ability to survive outside of a uterus, and all organs have not yet been formed.

## The use of hESC raises moral concerns

The moral status of the human embryo is a long-standing controversy thoroughly debated over the years. The ambiguity surrounding the status of the embryo has led to controversies without reaching a consensus [2]. Whether, and to what extent, the human embryo has a moral value that deserves protection or not, is repeatedly discussed alongside the medical development [3]. As more is learned about cells and their potential through medical research, the perceptions concerning the embryo, and the question of when human life begins, have been challenged. During the last few decades, this issue has become even more relevant as medical advances have shown that the embryo in the pre-implantation phase could potentially be used to produce medicinal products, to benefit patients and increase their health.

Human embryos are generated in regular medical practices for reproductive purposes for IVF procedures. Since the first successful IVF procedure, approximately 8 million IVF children have been born; at any given time point, it is estimated that more than 500,000 embryos are being kept frozen awaiting IVF procedures. After successful IVF procedures, embryos no longer considered for reproductive purposes are kept frozen in storage. The frozen embryos are considered for medical research or are destined for destruction [4].

In previous research regarding the moral dilemmas of using human embryos for non-reproductive purposes, the cryopreserved embryos have been described by IVF couples as a human being, a (potential) child or a sibling to already existing children [5–7]. Using embryos for non-reproductive purposes has been described by participants as callous [5]. A Swiss study from 2009 investigated the attitudes of IVF couples about the moral status of the embryo, revealing that 50% agreed that an embryo has the same dignity and rights as a human being [7]. Another study has shown that some consider their cryopreserved embryos as being too private to give away [8]. Individuals assigning embryos high moral status are less inclined to donate their embryos for research purposes [9]. There are also concerns about what will happen to the donated embryos; namely that the embryos will be misused or will be used to do “bad” things [5, 6]. However, previous studies show large variations in couples that donate, or would donate, for stem-cell research purposes. The least positive attitudes were captured in an Australian study (27%), while as many as 92% of couples at one Swedish IVF clinic consented to donate their surplus embryos for stem-cell research [10]. In one year, as many as 92% of the couples at one Swedish IVF clinic consented to donate their surplus embryos for stem-cell research [11].

In contrast, embryos are also described as “just a lump of cells” with no moral value, or that they gradually gain moral value as they develop [2, 7, 12]. According to this view, it may be acceptable to use hESC to develop a medical therapy. In previous studies, positive attitudes towards embryo donation are justified by the interest in helping others, making good use of embryos that otherwise would be discarded, wanting to give back to the society after having received medical care themselves, and wanting to contribute to medical advances [5, 8, 13]. A positive attitude towards embryo donation is associated with disagreement about the embryo having the dignity and rights of a human being [7]. Some describe that they do not want to waste the embryos, or the efforts needed to create them [14].

To our knowledge, there are no previous studies investigating patients’ views as potential receivers, but three studies have studied attitudes towards embryo donation specifically for medical treatment some years ago. The studies were performed with couples having undergone fertility treatment in China, Denmark and Switzerland. Specifically, 26–41% were positive towards donating embryos for treatment purposes themselves or suggested that embryo donation should be allowed [7, 15, 16].

### Induced pluripotent cells are not surrounded by the same moral issues as hESC

In parallel with the development of medical therapy using hESC, researchers have succeeded in producing induced pluripotent stem cells (iPS-cells) from human cells and using them for medical product development [17]. Unlike hESC, iPS-cells are derived from non-germ cells and thus are not surrounded by the same moral concerns Researchers are currently developing medical products to replace damaged or dead cells for various chronic diseases, e.g., for Parkinson’s disease (PD). Currently, the aetiology of PD is still unknown. There are no disease modifying therapies available for patients; rather, therapy focuses on symptom relief by compensating for low dopamine levels in the brain. Commonly, patients’ daily lives are increasingly affected over time by symptoms such as tremors, slow movements and balance problems. It is common to develop non-motor problems like depressive symptoms and, later, dementia. As the symptoms get worse with time, medicines are often given more frequently, and device-aided therapies are introduced. It is not uncommon for patients to suffer from side-effects of treatment, such as dyskinesia or behavioural problems.

Both iPS cells and hESC have demonstrated functional recovery in experimental PD studies, where iPS cells seem more tolerable, in addition to the reduced moral concerns [18]. There are mainly practical advantages of hESC over iPSC at the current stage of development for clinical applications. There are more hESC lines available that are of clinical trial grade (GMP, good manufacturing product) and a longer experience of the cell biological properties regarding safety and developmental stability over time. Given the extensive regulatory requirements for any medicinal product, most of the experience for this is with hESC products. IPSC from an individual with a genetic disease is unlikely to be suitable for an autologous application. The cost of production and also the regulatory requirements of such a product are very high [19]

Currently, the first studies are still ongoing and, except the one referred to, no results have been presented of any therapeutic effects of hESC in PD. One individual has received dopamine neurons from autologous iPSC, with some beneficial effects (1 h less off, and stabilised symptoms, with demonstrable surviving tissue in the brain, at 24 months [20]. Currently, there are 2 ongoing clinical trials; one in the US and Canada with a hESC cell product, and one in Japan with an allogeneic iPSC cell line, with another trial planned in Europe with a hESC derived product. No safety concerns have been reported to the authorities in the trials.

Oncogenic mutations have been reported in both transplant types. However, there is still debate on whether stem cell therapy is feasible, efficient and safe, and whether it affects enough of the PD neuropathology. Several methods have been developed to prevent tumour formation [20].

In summary, there is a lack of knowledge concerning values and preferences related to embryo donation for the development of medical therapies. We have not identified any studies investigating patients’ attitudes, as potential receivers, to embryo donation for production of medical therapy. What patients with PD think about medical treatment developed from hESC is still unexplored. Since they are significant stakeholders in the moral discussion presented, their views on the matter are arguably of great interest in itself as well as for policymakers and legislators.

### Aim

The aim of the study was to explore the views of patients with Parkinson’s disease (PD) on using hESC for treatment of PD.

## Methods

### Design

The study was a qualitative semi-structured interview study.

### Settings

In Sweden, it is legal to use left-over human embryos for research purposes but not to produce medical products. As of 2019, couples undergoing fertility treatments are allowed to cryopreserve their embryos for a maximum of 10 years before they must decide what to do with their surplus embryos. Surplus embryos can be discarded, donated to research, or donated to other couples and single women for reproductive purposes.

### Participant selection

Participants were consecutively sampled through two local PD patient organisations located in two Swedish cities. The heads of the patient organisations shared information about the study with their members (*N* = 377) by e-mail. Persons interested in participating or who had questions regarding the study were asked to contact the researchers. Seventeen individuals contacted the researcher, accepted participation and were interviewed. The participants were Swedish-born, had a mean age of 68.6 years (SD 9.6 years), and about two-thirds were male. They described their own religious backgrounds as either Christian, Evangelical, non-religious or atheistic. Additional participant characteristics are presented in Table 1.

**Table 1:** The characteristics of the participants (n = 17) presented with frequencies and percentages or mean (M) and standard deviation (SD)

	*n*/M	%/SD
Gender		
Female	5	29.4
Male	11	64.7
Other	1	5.9
Age	68.6	9.6
Born in Sweden	17	100
Occupation		
Working	5	29.4
Sick leave	2	11.8
Retired	11	64.7
Completed level of education		
Upper secondary school	4	23.5
Higher vocational education	2	11.8
College/University	11	64.7
Use of medication		
Daily	17	100
Time since PD diagnosis		
6–12 months	1	5.9
1–3 years	4	23.5
3–5 years	3	17.6
5–10 years	5	29.4
10–15 years	2	11.8
15–20 years	2	11.8

### Data collection

Due to the Covid-19 pandemic, the interviews were performed by telephone. The participants were free to participate from the location they preferred. Before the interviews, respondents received detailed informational material about hESC, and the potential treatment for PD was checked for accuracy by the neurologists in the research team. Informed consent was given by the participants, and they were asked to respond to a brief online questionnaire concerning their background characteristics. An interview guide was developed with input from representatives of the patient organisations and used for the semi-structured interviews (Table 2).

**Table 2:** Interview guide used for the semi-structured interviews

Can you please tell me what experience you have of medical treatments for Parkinson’s disease? (warm-up question)	
Have you previously heard of medical treatments using cells taken from embryos?	
If you recall when you first heard of medical treatments using cells from embryos, what were your first spontaneous thoughts?	
What is your view on using embryos for treatment of Parkinson’s disease? That donated embryos are destroyed in the process? That companies may profit from selling these medicinal products?	
In this question, what is important to you? Interests/values? To whom?	
What affects your outlook on this matter, do you think? Any beliefs?	
If you reflect on what you have told me so far, what aspects are most important to you concerning using surplus embryos to treat Parkinson’s disease?	
Have you changed your view on this matter during the interview?	
[Interviewer sums up what the participant has described so far]. Have I misinterpreted something, or do you want to add something to the summary?	
The purpose of this interview was to explore your views on using donated embryos for medical treatment and any values or interests related to it. Is there something you think of that has not been brought up yet?	

The interviews lasted between 32 and 105 min, with a mean of 58 min. Data collection was performed after approval was obtained from the Swedish Ethical Review Authority (Dnr 2019-06539).

### Analysis

The interviews were transcribed verbatim and analysed by three of the authors (JD, JVJ, EJ). The analyses were performed inductively using thematic content analysis, according to Burnard, Gill, Stewart, Treasure and Chadwick [21]. Shorter and longer text segments in the interviews were coded openly by writing briefly about what was being said by the participant. JD coded all the interviews, while JVJ and EJ coded five interviews each. One interview was separately coded by all three, and eight interviews were coded by two of the authors. The codes were compared, and differences were discussed and agreed upon. A list of all the codes was compiled, duplicates were removed, and similar categories were grouped together.

## Results

Three themes emerged while conducting the analyses: Factual beliefs and moral concerns related to the human embryo; moral positions concerning the use of hESC for medical treatment; and interests related to the use of hESC for medical treatment.

### The factual beliefs and moral concerns related to the human embryo

The philosophical question of when human life begins was described as decisive for the opinion regarding for what purposes human embryos should be allowed to be used. It was deemed impossible for a society to reach a consensus on when life starts. There were different perceptions of the moral status of the embryo. The embryo was described as being a mere lump of cells with the same value as any other cells. During the interviews, participants also likened the embryo to organs and germ cells. Some described the embryo as having no life. Thus, no life is terminated, and no one is hurt when embryos perish. Embryos were also described in terms of what they do not constitute; specifically, it was not seen as a child or a human being. One person described that the embryo has no life, as it lacks sensation. However, it was also looked upon as something special and as having a potential life. The human embryo was also seen as a resource, which is constantly produced in abundance. While some were firm in their perceptions, others were ambivalent and, at times, described the embryo inconsistently. Some explained that they would have had a different view of the embryo if it had been inside the womb or if it were older.

The embryo was ascribed no certain value and was not seen as something special by some participants.

Uhm, in some way, if I would have had it in, I mean if it was split in two fractions […] a tube of sperm wouldn’t be that exciting. […] But I am not convinced that it is much more exciting with a tube of eggs, and I am not convinced it is much more exciting if you mix them together. […] It becomes something else along the way, from being implanted and then, uhm, becomes a foetus, but when it is in the tube. […] The difference is not that dramatic, I mean. (Participant 17)

Moreover, some considered the embryo in terms of the equal value of all human beings, including embryos at a certain age of development.

We have agreement across all religions and life views that all human beings have equal value, but for a new human being, I set the somewhat arbitrary limit of eight weeks. (Participant 2)

Some ascribed a value to the embryo based on whether it will later become a human being or not. The embryo was considered to be highly valuable to the couple trying to become pregnant. However, when the embryo becomes superfluous, and the couple is no longer in need of it, its value was described as instrumental or as a means that could be useful for other purposes, such as helping others. The embryo was described as a valuable resource or a material.

I think it is a waste to throw these embryos away if they will not be used for assisted reproduction, in the same way as I think it is a waste not to use organs from brain dead individuals... which could save the lives of people who could live for many years with the transplant. (Participant 8)

In addition, some participants mentioned that embryos are discarded all the time, so they did not consider embryo destruction in itself to be problematic. Embryos are, by nature, not used and destroyed all the time, and they do not need to be protected for their own sake. One person thought it was absurd to consider the embryo as human life with human rights. In contrast, the embryo was also seen as something intrinsically special that cannot be likened to a commodity or just thrown away. Because of its potential, participants assigned a certain value to the embryo; therefore, the use of embryos for medical treatments needed a justification from an ethical perspective.

### Moral positions concerning the use of hESC for medical treatment

Participants’ moral positions regarding the use of embryos for therapy development were diverse. It was perceived as a complex and difficult issue that some individuals had never thought of, and it triggered thoughts and feelings. One person asked herself how much one should change the course of nature.

How far should we go in changing [nature], as in genetics, uhm, where do we draw the line on what is ethical, that is what I think about a lot... (Participant 13)

Some had no firm opinions, while others were clear about their positions. More knowledge of the effects of the treatment was important for some to make a decision. Some thought it should be up to the experts, researchers (with no interest or profit) or legislators to decide whether the embryos should be used for medical purposes.

It is up to the researchers to use them in a right way...one has to follow the current legislation. I have no concerns in my conscience regarding this. (Participant 4)

There were some negative reactions after learning about the treatment. Some found it scary initially or became upset, but these reactions subsided when they realised that cryopreserved embryos, and not foetuses, are used. Others described having cautiously negative initial attitudes. Some were concerned about how the embryos would be used and handled. One person described that it felt wrong to destroy an embryo that could have developed into a human being. Some assigned their negative attitude towards it being a new treatment, not having more information about the treatment or always being reserved when it comes to new treatments. Not understanding why embryos need to be used made one participant suspicious.

So my first [thought] was that I will not say it was all negative, but it was in the negative direction; this feels a bit tricky. What are they going to do with it? What are they after? I don’t understand. (Participant 13)

However, overall, the participants were positive towards the use of hESC for treatment of PD. They found the treatment interesting and exciting and thought that it should be used if it could be beneficial to others. Using surplus embryos for medical product development was seen as something positive, “natural” or heart-warming, as it increases their utility, compared to discarding them. This was regardless of whether iPS-cells were also available for treatment. One participant expressed that it was simple and intuitively easy to understand the potential of hESC. An individual who had previously received a transplant claimed it would be presumptuous of him not to accept this treatment based on his medical history. Using hESC was described as unproblematic. One person explained that no one’s integrity is threatened. Another said it was unproblematic to use embryos to repair or reduce damage, but not to improve the human being.

In this case, a person with Parkinson’s has lost something existing; if you can replace it in this way, then I don’t see an ethical problem with it at all…

…I find it harder to accept that you, in some way, improve nature, but replacing what has been lost is not problematic at all to me. (Participant 14)

The participants were positive towards the idea of receiving treatment with cells coming from embryos themselves; some said they would accept treatment if it was offered to them. They saw the treatment as a potential help to themselves in the future. One participant said that he would donate any embryos he had for medical treatment if he was able to.

Some thought that the effects of hESC and iPS-cells should determine which of the treatments to use. The treatment that is best should be developed. Meanwhile, some thought that only iPS-cells should be used if there is a choice. Such cells were preferred since they avoid the ethical issues surrounding hESC and also because iPS-cells are more easily available. Others preferred the use of hESC over iPS-cells. One person stated it was preferable to use embryos instead of skin cells from an ethical point of view, as someone is hurt when sampling skin cells. Embryos were perceived as more appropriate, based on how they are to be used; the cells have never been specialised, they are not as old, and were believed to have a better treatment effect than iPS-cells.

### Interests related to the use of hESC for medical treatment

The participants identified several interests related to the use of human embryos for development of medical therapies. They balanced the interests they identified against each other during the interviews. Their interests concerned not only them as patients but also brought up interests of others.

#### Interests relating to themselves as patients

The participants described an interest in having a treatment that did not limit an active everyday life, and that increased their health and gave them a better life with higher quality. An easy everyday life was desirable, without any tubes or cords attached to them as well as not having to take multiple doses of medicines every day. Some described that learning about this therapy gave them hope of a better and longer life for themselves and/or for other patients. Participants hoped that the treatment would be used early on and that it would slow down the progression of the disease, but they also saw it as a potential cure for PD. They hoped that undergoing treatment would prolong life and reduce pain, suffering and other PD symptoms. Some were interested in increasing their functions, e.g. cognitive functions, communication abilities, mobility, being able to help relatives, and they wanted to be more independent in their everyday lives. One participant described how he wished that he did not have to wake up his wife every night to help him get to the bathroom. Another participant wished for persons with PD to be able to return to a normal life again. Development of new treatment alternatives as well as these potential benefits for patients and relatives were used to justify the use of hESC.

The participants expressed a need for improved medical treatments against PD. Some described that the medicines they had tested so far had no, or insufficient, effect. The efficacy of the treatment was described as important, and there were concerns that these treatments would not be efficacious enough.

Patient safety was important, and participants worried about potential side-effects of the treatment. Injecting substances into the brain was perceived as something risky. Some were solely worried about having to undergo a brain surgery; others were concerned about the substance and its short- and long-term side-effects.

…what happens in the body [and] what happens in the long run in the next generation, is it something you carry with you… everyone who has Parkinson’s is not 78, but there are some that get Parkinson’s very early on.

If they then have children, what does … this treatment do to the next generation. (Participant 13)

Participants also worried about cell rejection, becoming ill, the cells being put in the wrong place or losing their functions, such as limiting the ability to go biking or swimming.

It was important for them as patients to be treated with respect, get individualised treatment and to decide their treatment together with their doctor. They wanted to be properly informed about the treatment alternatives beforehand and were interested in learning what the treatments entailed and how they are manufactured. This information would help them to decide whether or not they would accept treatment. However, some participants did not think it was important to know where the embryos came from. As patients, they were not only interested in receiving proper information about available treatment alternatives but also about research and medical advances being made within the area. This new knowledge gave them hope, not only for themselves, but also for the sake of their children and future generations.

#### Patients’ interests related to the donors

It was important for the participants that the donating woman or couple were donating embryos voluntarily. Specifically, donors should be able to choose if and for what purpose they want to donate, and they need to give their consent. However, one person thought it was not necessary to have the couple’s consent as long as it is clear that the couple no longer wants them. Some described it as being important that the couple got to decide what to do with their embryos without coercion and that they and their decision should be respected. The embryos were described as theirs, and they should therefore decide over them.

Some feared that women or couples will be used or exploited in the process. It was speculated that an increased embryo demand could possibly lead to a black market, where embryos are traded. One person feared that an increased demand could possibly lead to pregnancies being imposed and ended against women’s will in some parts of the world. There were also concerns that being able to sell embryos and get financial compensation would give couples incentives to donate embryos against their own will. Already poor and vulnerable groups were seen as being at risk of being exploited. There were disagreements when it came to economic compensation for donors. A “thank you” was suggested to be compensation enough, while others thought it was reasonable to provide financial compensation to the couple. However, participants had difficulties in setting a reasonable amount for compensation. Participants thought that donors deserved to be respected throughout the process and be well taken care of, as well as shown appreciation and gratitude. By only allowing donation of surplus embryos from fertility treatments, one would be more confident that the primary purpose of creating the embryos was to have a child and not to earn money.

#### Patients’ interests related to the society

Participants acknowledged there being various attitudes in society regarding using embryos for medicine development. It was seen as desirable to have a joint view on this matter in society. It was deemed important to discuss the issue openly, and efforts should be made to reach a consensus. Participants felt that at least most people should find it acceptable.

It is very good that this issue is being discussed because I think it is important that we have some level of agreement, and there are different value systems that lead to different conclusions. But for me, I only see advantages. (Participant 16)

Embryo destruction was expected to upset people, considering the embryo to be life or life in being. There was an understanding of the different attitudes in society and the resistance towards using embryos for this purpose. Public debates and headlines in the newspapers were foreseen. Some expected negative attitudes based on how, e.g. abortion had been debated in some countries. They expected people to have different views for personal, ethical and religious reasons, and that some would refuse to handle the embryos for reasons of conscience. It was suggested that different opinions should be met with respect.

Before deciding upon this matter, it was expressed that the question deserves careful considerations and an investigation of people’s attitudes and feelings in relation to making treatment available. It was also suggested that new legislation be written taking into consideration present and future, long-term, consequences. Specifically, what will happen to embryos that are donated but not used? Another participant pointed out that with medical advances being made continuously, any changes in the legislation might unintentionally allow us to do things in the future that one is not capable of doing, nor aware of, today. Consistency in the legislation on what you can use embryos for was considered important.

Some participants described a lack of trust in the process of producing medicines. There were concerns regarding researchers and pharmaceutical companies not having an ethical compass and that they would not handle the embryos in an ethical manner. Some thought that the embryos should be handled carefully and with respect. However, some also suggested that they did not need to be treated in any special way. There were concerns that the pharmaceutical companies could withhold treatment from patients to increase the demand and the prices. Participants stated having higher trust in public organisations and thus preferring to have them be responsible for medical development. Information and transparency towards the general public about how treatment is developed, its *pros* and *cons*, explaining why some embryos are used and not others, and that there is an oversight in place, were important aspects for participants.

I have no trust in companies working with welfare, such as health care and schools. I don't trust that they will use embryos in an ethical way. (Participant 13)

Making profits on treatments developed using cells from embryos was unproblematic for some, whilst others thought it was problematic or “wrong”. Some did not see it as a problem as long as no one was being fooled. The industry’s top priority should be patients’ health, benefit to the general public and making the treatment available for patients, not to profit from them or their illness. They were afraid that money could steer the direction of medical development. Some saw profiting as a problem when it restricts availability for patients, or when there is profit arising from publicly financed research. Low costs for the patients were desired. Some saw it as more problematic to earn money on cells coming from an embryo compared to other medicines, while others felt it did not make any difference. It was important that patients’ health was always prioritised more than profit-making in the industry. Some participants preferred non-profit organisations to produce the therapy, but it was also seen as an unrealistic scenario.

Public health and having healthy citizens were valuable points for participants. There were also concerns that treatment with cells from embryos would increase the societal and global injustice. Participants wondered which patients the treatment would be available to. Medical care was seen as a human right, and participants wished for it to be available for all patients. It was believed that the pharmaceutical industry’s involvement and high prices of the therapy would limit the availability of the treatment for patients who are in need. Participants believed that patients with the greatest needs might not even be able to receive treatment in some parts of the world. In other parts, the prices might be too high and lead to only people with the greatest needs or wealth being able to use it. Participants requested a fair prioritisation between patients. Some suggested that the ones with the most severe condition should be prioritised because they have a greater need, whilst others suggested that younger patients should be prioritised because they are easier to help and still contribute to the society by working.

The national economy and reduction of treatment costs were seen as important and should influence the decision on whether to use hESC or iPS-cells. Using both hESC and iPS-cells in therapy development was seen by others as positive, motivating why it was unnecessary to limit one’s options early on when not knowing their full potential. Participants felt that such treatment could help reduce healthcare costs and increase patients’ ability to work.

## Discussion

As described in the background, there are various moral concerns among ethicists, lawyers and policymakers related to the use of human embryos for non-reproductive purposes. Regarding purposes related to medical treatment, it is of special interest to explore the attitudes of patients who are seeking safe and effective treatment. They are directly concerned stakeholders, in the sense that policymaking and legislation may directly affect the possibility of improved treatment. Patients need to be informed about and consent to any treatment proposed to them by their treating doctor. The information needs to be sensitive to the special needs and concerns of the patient. In a field where there is an ongoing moral discussion in society, such as in using hESC for medical therapy, a better understanding of patients’ attitudes is therefore of particular relevance. It can be of help for clinicians who are expected to provide meaningful information, but also for policymakers and legislators. It may also guide the thinking and planning for other areas of cell therapy, which is a rapidly growing field in medical science. This is the first study that explores patients’ views on using hESC for medical treatment. It reveals a wide spectrum of disclosed factual beliefs and moral concerns related to the human embryo, moral positions concerning the use of hESC for medical treatment, and interests related to the use of hESC for medical treatment. The participants had a positive or ambivalent attitude towards the use of hESC in the treatment of PD. Interests relating to themselves as patients, to the donors and to the society were identified and were important for the patients to take into account.

Generally speaking, patients did not consider the human embryo to constitute human life, but it was also seen as something special. The participants accepted hESC being used in treatment, although some were hesitant. It is reasonable to believe that their moral positions are consequences of their views on superfluous human embryos as not representing human life in need of protection. Profiting from developing treatments using hESC was seen as reasonable by some, while others found it problematic. There were concerns that the industry prioritises profit over patients’ well-being. Transparency is important to establish trust in the industry and researchers, and public debates respecting all opinions were important to the participants.

There are no previous studies on patients’ views on using hESC for medical treatment, but if you compare the results to previous research performed on couples, differences may be found. Previous studies report individuals claiming that human embryos have the same dignity and rights as humans [2, 7]. This was not found within our data collection. However, some time has passed since those studies were performed, and they were conducted within other study populations. It is expected that patients, as potentially being the ones that benefit from the treatment, would be more positive towards it compared to other groups. They have experienced living with the disease and not having the desired effects from their treatment as well as a lack of treatment. In addition, this research was performed in Sweden, one of the first European countries to allow stem cell research [22] Previous research has indicated Swedes as having positive attitudes towards the use of superfluous embryos for non-reproductive purposes. Bjuresten and Hovatta [11] showed that more than 9 out of 10 couples approached chose to donate their surplus embryos to stem cell research. Thus, the results indicate that Swedish patients with PD would accept treatment with hESC.

Regarding donation and use of left-over human embryos, there are some similarities with ethical issues raised in the context of organ donation for transplantation. In one of the quotes above, the participant compared embryo donation for medical use with the use of organs from brain dead individuals. In both cases, the issue of consent was raised, and an informed consent from the couple is seen as a natural requirement. Taking organs from a human body also requires an expressed consent. When the will of the person is not known, regulatory frameworks differ regarding the role of relatives, who, in some cases, have a right to veto a decision to donate. However, many countries have moved towards a policy of presumed consent [23]. One may assume that clinicians would still be reluctant to go against the will of relatives of the deceased. In biobanking, as in organ transplantation, there is an agreement regarding the prohibition of commercialisation [24, 25] Human embryos may not be sold, but there are commercial interests involved in the development of stem cell derived therapies, and patenting may be seen here as a legitimate interest [26, 27].

The results here should be interpreted with caution. It is likely that our results do not present the full picture. It cannot be excluded that some views have not been captured due to the limited number of patients enrolled in this qualitative setting. The absence of foreign-born individuals and the high percentage of highly educated participants support that theory. It may also be the case that mostly individuals with already strong convictions decided to participate, therefore not providing a full picture of the various concerns and interests. It should also be observed that this study only reflects the views of the participants, a selected group of patients assumed to have a special interest in the improvement of treatment opportunities. One cannot expect other groups to have similar opinions. The findings are in agreement with the results of a recent interview study with the general population [28]. However, there is also a need for more research, including the use of quantitative methods, to study current views and attitudes of patients and other relevant groups as IVF-couples and the general public, as values and moral positions can change over time and with medical advances being made.

## Conclusion

There is a wide range of concerns among patients with Parkinson's disease revealed in this study. It may help clinicians and researchers developing cell-based treatments to be more sensitive to patients’ needs and concerns. Overall, there is support for patients’ acceptance regarding the use of hESC for medical treatment, since the embryo is not seen as constituting life to be protected by human rights. However, this needs to be followed up in quantitative studies with a larger number of patients. There are some concerns that the industry will not always prioritise the patient over profit. Transparency is therefore seen as vital. Science, medical advances and healthcare depend on trust from society and ultimately on how development of new treatments affects patients. It is therefore important to take patients’ views seriously and consider these when deciding on how human embryos are allowed to be used.

## Data Availability

Supporting interview data are available upon reasonable request from the corresponding author.

## Abbreviations

hESC: Human embryonic stem cells
IVF: In vitro fertilisation
ATMP: Advanced therapy medicinal product—product on the market for treatment of human disorders
iPS-cells: Induced pluripotent stem cells
PD: Parkinson's disease
